# Development of a genotype independent and transformation amenable regeneration system from shoot apex in rice (*Oryza sativa* spp. *indica*) using TDZ

**DOI:** 10.1007/s13205-012-0051-y

**Published:** 2012-02-28

**Authors:** Mohitosh Dey, Souvika Bakshi, Gabor Galiba, Lingaraj Sahoo, Sanjib Kumar Panda

**Affiliations:** 1 Department of Biotechnology, Indian Institute of Technology Guwahati, Guwahati, 781039 Assam India; 2 Department of Life Science and Bioinformatics, Assam University, Silchar, 788011 Assam India; 3 Department of Plant Molecular Biology, Agricultural Research Institute of the Hungarian Academy of Sciences, 2462 Martonvásár, Hungary

**Keywords:** *Agrobacterium*, *Indica* rice, Plant regeneration, Shoot apices, Thidiazuron

## Abstract

*Agrobacterium*-mediated transformation of *indica* rice has been established in only a limited number of cultivars because the regeneration of plants from transformed embryogenic calli is highly cultivar-specific. Establishment of a highly efficient plant regeneration system from shoot apex explants applicable to many cultivars of *indica* rice will accelerate the application of transformation technology in breeding programs and functional genomics study. We established an efficient shoot multiplication and plant regeneration system from shoot apices of *indica* rice using thidiazuron (TDZ) as a plant growth regulator. Shoot apices cultured on MS basal medium devoid of plant growth regulators formed solitary shoots in 90% of cultures. Addition of TDZ or benzylaminopurine to regeneration medium significantly influenced formation of multiple shoots directly from shoot apex explants without an intervening callus stage. Best shoot proliferation response (10.3 shoots per explant) was recorded when shoot apices were cultured on media supplemented with 4 mg/l TDZ. No synergistic effect on shoot proliferation was observed when indole-3-acetic acid and indole-3-butyric acid were supplemented to media containing 4 mg/l TDZ. The regeneration system was efficient in evoking multiple shoot proliferation in eight different cultivars of *indica* rice. Shoots were rooted in MS basal medium and plantlets were acclimatized with 100% survival rate. The shoot apex explants of all the eight cultivars of *indica* rice were found competent to *Agrobacterium*-mediated transformation while explants from IR-64 showed highest transient GUS expression. This variety-independent transformation amenable regeneration system from shoot apices may widely be applicable for genetic transformation of *indica* varieties.

## Introduction

Rice (*Oryza sativa* L.) is the main staple food for more than half of the world population. Around 80% of the world rice production is based on *indica* varieties, which are grown under subtropical and tropical conditions as long grain rice, and thus securing a unique position in agriculture (Khush 1997). It has also become a model monocot system for genetic and functional genomic studies (Jung et al. 2008). In recent years, considerable progress has been made in the improvement of important agronomic traits of rice through biotechnological approaches (Hao et al. 2009; Skamnioti and Gurr 2009). Genetic transformation has become an important tool in targeted improvement and gene function studies in rice (Xu et al. 2012). Most plant regeneration systems adapted to *Agrobactrerium*-mediated genetic transformation in *indica* rice varieties (Rashid et al. 1996; Aldemita and Hodges 1996; Nayak et al. 1997; Zhang et al. 1997; Khanna and Raina 1999, 2002; Mohanty et al. 2002; Supertana et al. 2005; Ignacimuthu and Arockiasamy 2006) involve regeneration of plants from transformed embryogenic calli, anther calli (Jiang et al. 2004), and protoplasts. Success in transformation of *indica* rice using such regeneration systems depends on the factors that favor the formation of friable and high quality callus in a shorter time competent for shoot regeneration. The potential for callus induction and regeneration have been reported to be variety-dependent, limiting efficient regeneration in large number of regional *indica* rice varieties for genetic manipulation (Ali et al. 2004). Moreover, identification of callus amenable for transformation is cumbersome and the regeneration process is time consuming. Furthermore, inflorescences and immature embryos are available only for a limited period in a year because of photoperiodic sensitivity of rice genotypes. Quick loss of regeneration potential in calli, several stages of subculture to select the transformed calli, and problems associated with isolation and sterilization of immature embryos are the serious limitations for use of these explants in transformation (Kishore et al. 2006).

Use of shoot apex for successful genetic transformation through both *Agrobacterium* and biolostic methods is reported in many cereals including rice (Sticklen and Oraby 2005). A major advantage with shoot apex for genetic transformation is its developmental plasticity which allows rapid and direct regeneration of transgenic plants from transformed shoot apices ensuring cultivar integrity and circumventing the appearance of cell culture induced mutations (Hirochika et al. 1996; Bao et al. 2001). Manipulation of transgenic meristematic cells in shoot apices by treatment with growth regulators for induction of multiple shoots is most attractive for generation of stable transformants. Thidiazuron (TDZ), a phenylurea-type cytokinin, has been reported to facilitate efficient multiplication of apical meristem cells and their reprogramming to appropriate developmental stage for shoot differentiation (Gairi and Rashid 2004; Goldman et al. 2003; Srivatanakul et al. 2000). However, to our knowledge, plant regeneration from shoot apex is available only for two varieties, White Ponni (WP) and Pusa Basmati 1 (PB1) of Indian origin (Arockiasamy and Ignacimuthu 2007) of *indica* rice.

We report establishment of an efficient plant regeneration system from shoot apices of *indica* rice applicable to eight cultivars using TDZ as a growth regulator and demonstrate their amenability to *Agrobacterium*-mediated transformation.

## Materials and methods

### Plant material and explant preparation

Seeds of eight *indica* rice cultivars IR-64, Anjali, Vandana, Chandan, Mahasuri, Nilagiri, Ranjit, and Luit having superior attributes including good grain quality and varying levels of disease resistance were obtained from Regional Rainfed Low Land Rice Research Station, Gerua, Assam, India. The cultivar IR-64 was used as a model for *indica* rice shoot apex regeneration and transformation experiments. Mature seeds were dehusked, surface sterilized with 70% ethanol for 30 s, rinsed with 1% bavistin, and 0.2% HgCl_2_ (w/v) for 5 min each. The seeds were then rinsed five times with sterile double distilled water and cultured on MS basal medium (Murashige and Skoog 1962) with 3% sucrose supplemented with or without 1 mg/l TDZ. Shoot apices (4–5 mm) were carefully excised from the four-day-old germinated seedlings and used for all experiments.

### Multiple shoot induction and plant regeneration

In order to study the effect of most potential cytokinins, benzylaminopurine (BAP) and TDZ on multiple shoot induction, shoot apices of *indica* rice cultivar IR-64 were cultured on MS medium supplemented with different concentrations (1, 2, 3, 4, and 5 mg/l) of TDZ or BAP. The regenerating explants were subcultured twice onto fresh media at an interval of 15 days each. The efficiency of multiple shoot induction and plant regeneration were evaluated by scoring the mean number of shoots induced from responding explant, and measuring the mean shoot length. Regeneration frequency was calculated based on the number of shoot apices responding to regeneration by total number of shoot apices cultured.

The synergistic effect of auxin with 4 mg/l TDZ on shoot proliferation was examined by supplementing different concentrations (0.025, 0.1 and 0.25 mg/l) of indole-3-acetic acid (IAA) or indole-3-butyric acid (IBA) to MS medium containing 4 mg/l TDZ. The regenerating explants were subcultured following the methods described earlier. Percentage of regeneration, mean number of shoots, and average shoot length were recorded. All media were adjusted to pH 5.8 with 0.1 N NaOH or 0.1 N HCl prior to addition of 0.7% agar–agar (Hi-media, Mumbai), and autoclaving at 20 psi and 121 °C for 20 min.

### Effect of genotype

The effect of genotype on multiple shoot induction and plant regeneration from shoot apices was studied by culturing shoot apex explants of *indica* rice cultivars, Anjali, Vandana, Chandan, Mahasuri, Nilagiri, Ranjit, and Luit in MS medium supplemented with 4 mg/l TDZ. The number of regeneration responsive explants, mean number of shoots, and average shoot length were recorded. The relative frequency of plant regeneration and efficiency of multiple shoot induction were compared among all cultivars.

### Rooting and transplantation

The shoots (5–7 cm) were separated from multiple shoot clumps and transferred to MS medium devoid of growth regulator for 2 weeks for root formation. Subsequently plantlets with well-developed root system were washed in tap water and acclimatized in polybags containing soil and vermicompost (1:1), covered with transparent polybags at 27 °C and 16 h photoperiod for 14 days. Finally, the hardened plants were transferred to pots containing soil and established in a green house.

### Culture conditions

All cultures were maintained under the same experimental conditions at 25 ± 2 °C under white fluorescent light at irradiance of 37.5 μmol/m^2^/s with 16-h photoperiod. Visual observations of the cultures were taken every week, and the percentage of cultures showing regeneration, number of shoots per explant, and shoot length were recorded after 30 days.

### Transformation procedure and GUS assay

**Fig. 1:**
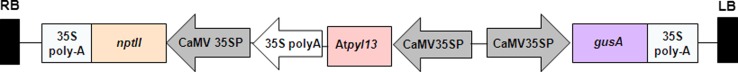
T-DNA region of pCAMBIA2301*Atpyl13* (12 kb). The 495 bp (*Eco*RI–*Hin*dIII) fragment containing *Atpyl13* under control of CaMV35S promoter and *NOS* terminator. *LB* and *RB* left border and right border of T-DNA region, *NOST* nos terminator, *35P* CaMV35S promoter, *nptII* neomycin phosphotransferase II

*Agrobacterium tumefactions* strain EHA105 harboring a binary vector pCAMBIA2301*pyl13* which contains a ABA receptor gene (*pyl13*), β-glucuronidase (*gus*) interrupted with an intron in the coding region and neomycin phosphotransferase (*nptII*) genes, all driven by CaMV35S promoter (Fig. 1), was used for transformation studies. The bacteria was grown on YEP (10 g/l yeast extract, 10 g/l peptone, 50 g/l NaCl, 15 g/l agar–agar and pH 7.0–7.2) solid medium containing 50 mg/l kanamycin and 10 mg/l rifampicin at 28 °C. A single bacterial colony was inoculated into 2 ml of liquid AB medium containing 5 mg/l rifampicin and 25 mg/l kanamycin and grown overnight on a rotary shaker at 200 rpm at 28 °C. Bacteria were pelleted at 5,000 rpm for 5 min and resuspended in liquid MS medium containing 100 μM acetosyringone at a density of OD_600_ = 1. Shoots apices excised from 4-day-old seedlings were gently stabbed four to five times using a sterile needle (24 G) at apex region before being immersed in bacterial suspension for 30 min with shaking at 80 rpm at 25 °C. Inoculated explants were blotted on sterile filter paper and co-cultivated on solid MS medium containing 1 mg/l TDZ and 100 μM acetosyringone for 3 days at 25 °C under dark condition. After co-cultivation, the explants were washed three to four times with sterile double distilled water by vigorous stirring, blotted dry on sterile filter paper and 20 explants were tested for transient *gus* expression by histochemical assay using 5-bromo-4-chloro-3-indolyl glucuronide (X-Gluc) as a substrate (Jefferson et al. 1987). The explants were then visually scored for transient *gus* activity.

### Regeneration of transgenic plants

Following co-cultivation, the explants were washed three to four times with sterile double distilled water by vigorous stirring, blotted dry on sterile filter paper and were cultured on selection medium (MS medium containing 4 mg/l TDZ, 45 mg/l kanamycin, and 500 mg/l cefotaxime) for induction and selective regeneration of transformants. The cultures were transferred to fresh selection medium at an interval of 15 days. Same levels of antibiotics were maintained during subsequent subcultures. After 4 weeks of culture on selection, the proliferating kanamycin resistant shoots (>5 cm) were transferred to rooting medium (MS medium containing 500 mg/l cefotaxime). The putative transformed plants were established in soil:compost (1:1) and grown to maturity in transgenic greenhouse containment.

### Molecular analysis of transformed plants by PCR

Genomic DNA was isolated from the leaves of transformed and untransformed (control) plants by modified CTAB method (Solleti et al. 2008). PCR analysis was carried out for *gus* gene to amplify 570 bp internal fragment using 18 mers (*gus* Fw: CTGTGGGCATTCAGTCTG; Rv: ACGCTGACATCACCATTG) primers. To rule out the possibility of *Agrobacterium* contamination, PCR was performed to check for amplification of 760 bp fragment of the bacterial virulence gene (*virG*) located outside the T-DNA using 21 mers (Fw: ATGGCTGGCCAGGATCCTAGA; Rv: TCAGGCCGCCATCACACC) primers. The amplification reaction was carried out under the following conditions: 94 °C for 5 min (1 cycle), 94 °C for 1 min (denaturation), 58 °C for 1 min (annealing), 72 °C for 1 min (extension) for 35 cycles followed by the final extension at 72 °C for 7 min (1 cycle). PCR was performed using ~100 ng of purified genomic DNA, 50 ng plasmid DNA (pCAMBIA2301*Atpyl13* as positive control), and Taq DNA polymerase (Genei, Bangalore, India) according to manufacturer’s instruction. The negative and untransformed plant controls were set up with no DNA and untransformed rice plant DNA, respectively. The amplified products were resolved by electrophoresis on 1% agarose gel and visualized by ethidium bromide staining (Sambrook et al. 1989).

### Statistical analysis

Data were subjected to analysis of variance (ANOVA) and mean separation by Duncan’s multiple range test (DMRT) using single-factor completely randomized block design to study the effect of different treatments on shoot proliferation and frequencies of transient expression. All experiments were performed at least three times with a minimum of 30–40 explants per treatment.

## Result and discussion

A prolific regeneration system based on multiple shoot induction from isolated shoot apices permits quicker plant regeneration owing to their extensive proliferative ability and presents amenability to germline transformation. Furthermore, use of shoot apices from in vitro germinated seedlings facilitates availability of explants round the year. We developed an efficient and reproducible plant regeneration system from isolated shoot apices of *indica* rice by use of a synthetic urea-cytokinin, thidiazuron (TDZ). The regeneration system was found applicable to eight *indica* rice cultivars investigated in this study and amenable to *Agrobacterium*-mediated transformation.

### Effect of TDZ on shoot multiplication

**Fig. 2:**
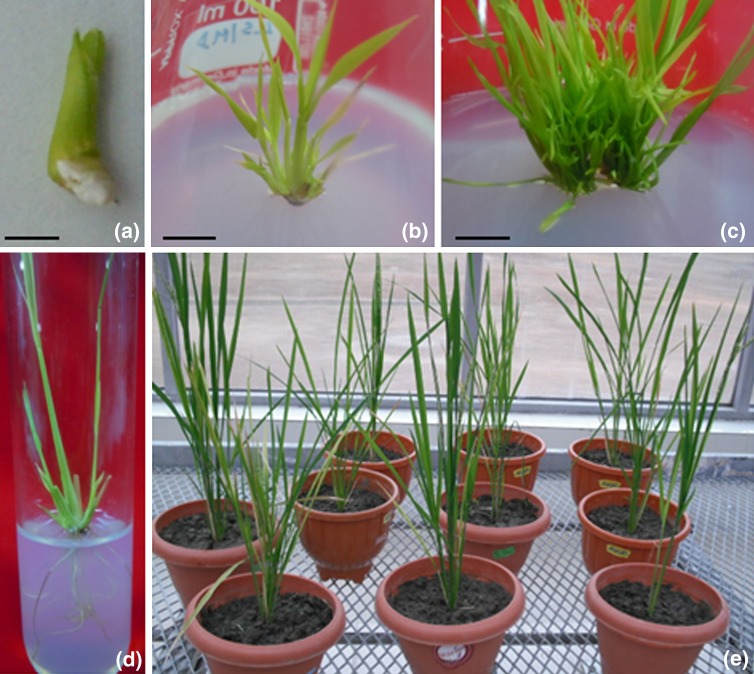
Multiple shoot induction and plant regeneration from apical meristems of *Oryza sativa* spp. *indica* cv. IR-64*.***a** Shoot apex explant. **b** Induction of shoots from shoot apex in MS medium supplemented with 4 mg/l TDZ after 2 weeks in culture. **c** Shoot proliferation from shoot apex in MS medium supplemented with 4 mg/l TDZ after 4 weeks in culture. **d** Rooted shoot in MS medium. **e** Regenerated plants growing in greenhouse. *Bar* represents 2 mm (**a**); 1 cm (**b**); 2 cm (**c**); 2 cm (**d**)

The morphogenic potential of shoot apices of the eight *indica* rice cultivars was analyzed on MS medium augmented with various concentrations of TDZ and BAP. The efficiency of shoot induction from shoot apices on growth regulator free MS medium was considered as control. Shoot apices (Fig. 2a) cultured on MS medium formed a solitary shoot without callus formation at the base in 90% of responding explants (Table 1) within 2 weeks of culture. However, inclusion of various concentrations (1–5 mg/l) of TDZ and BAP in basal medium elicited a prime and distinct role in shoot buds differentiation within 2 weeks of culture. One of the main functions of cytokinin is known to confer morphogenic competence for initiation of shoot proliferation. However, the type and concentration of cytokinin influenced the regeneration frequency, average number of shoots produced per explant, and mean length of the shoots (Table 1). Although, addition of both TDZ and BAP into medium resulted in induction of multiple shoots, but the effect of TDZ was more pronounced than BAP at equimolar concentrations (Table 1). Of the different concentrations of TDZ and BAP tested, 4 mg/l TDZ was found to be most effective in inducing multiple shoot induction from the shoot apices by producing maximum of 10.3 shoots per explant in 89% of cultures (Table 1; Fig. 2b, c). A linear correlation between increases in TDZ concentration to an optimal dose (4 mg/l) and regeneration frequency as well as mean shoot number was recorded (Table 1). Thidiazuron, a phenylurea-based compound has shown to possess potent activity as a cytokinin in inducing efficient multiple shoot formation in several plant species (Huetteman and Preece 1993, Srivatanakul et al. 2000; Mithila et al*.*^2001^; Goldman et al. 2003; Gairi and Rashid 2004; Faisal et al. 2005; D’Onofrio and Morini 2005; Radhika et al. 2006; Siddique and Anis 2007). Higher concentration of TDZ (5 mg/l) resulted in a decrease in regeneration frequency, mean shoot number, and shoot length indicating suppression of shoot proliferation (Table 1). Furthermore, stunted shoot growth was recorded at 5 mg/l TDZ as the shoot buds appeared to be developmentally suppressed. Unlike BAP, an adenine and purine based cytokinin, TDZ is resistant to cytokinin degrading enzymes, and therefore at high dose remains persistent in the tissues inducing excessive suppression of shoot buds, consequently leading to reduced proliferation rates (Huetteman and Preece 1993). In comparison to TDZ, shoots induced on BAP containing medium were longer and slender.

**Table 1:** Effect of different cytokinins on shoot multiplication and plant regeneration from shoot apices of *Oryza sativa* spp. *indica* cultivar IR-64 on MS medium after 4 weeks of culture

Cytokinin concentration (mg/l)	Regeneration (%)	Mean shoot number	Average shoot length (cm)
TDZ			
0	90^a^	1.0 ± 0.02^e^	10.0 ± 0.25^a^
1	65^c^	4.0 ± 0.14^c^	9.5 ± 0.47^b^
2	70^c^	4.6 ± 0.38^c^	9.0 ± 0.54^b^
3	81^b^	8.5 ± 0.19^b^	8.0 ± 0.44^c^
4	89^a,b^	10.3 ± 0.20^a^	7.0 ± 0.43^c,d^
5	60	8.3 ± 0.50^b^	4.0 ± 0.45^f^
BAP			
0	90^a^	1.0 ± 0.02^e^	10.0 ± 0.25^a^
1	62^c,d^	2.4 ± 0.17^d^	9.3 ± 0.20^b^
2	52^d^	1.2 ± 0.38^e^	7.5 ± 0.23^c^
3	50^d,e^	1.6 ± 0.14^d,e^	6.5 ± 0.22^d^
4	43^e^	2.0 ± 0.24^d^	5.6 ± 0.32^e^
5	57^d^	2.3 ± 0.30^d^	5.7 ± 0.27^e^

Values represent mean ± SE

Mean values followed by the same letters are not significantly different by the Duncan’s multiple range test at *p* = 0.05

Effective multiple shoot induction and shoot proliferation is often the manifestation of interactions among physiological state of the explants and a combination of plant growth regulators (Mallikarjuna and Rajendrudu 2007). In our study, we evaluated the effect of different concentrations of IAA and IBA in combination with optimal dose of TDZ (4 mg/l) on enhancement of multiple shoot induction from shoot apices. However, no incremental increase in regeneration frequency as well as mean shoot number was observed indicating the absence of synergistic effects of IAA and IBA with TDZ on cultured shoot apices of *indica* rice cultivars in our study (Table 2). Our results are contrary to the report on effective multiple shoot formation through the use of a combination of cytokinins, TDZ and BAP with auxins in immature embryo of another cereal, sorghum (Pola et al. 2007; Kishore et al. 2006). Hormonal metabolisms are known to be operated in an integrated manner (Gaspar et al. 2000) and that several, potential, mutual functional interacting points exist between different hormones (Coenen and Lomax 1997). Both synergic as well as antagonistic effects among plant growth regulators have been reported in effecting shoot proliferation in in vitro cultures.

**Table 2:** Effect of different auxins in combination with 4 mg/l TDZ on shoot multiplication and plant regeneration from shoot apices of *Oryza sativa* spp. *indica* cultivar IR-64 on MS medium after 4 weeks of culture

Auxins (mg/l)	Regeneration (%)	Mean shoot number	Average shoot length (cm)
IAA			
0.025	71^c^	4.3 ± 0.16^b^	7.0 ± 0.33^b^
0.1	77^a^	5.0 ± 0.19^a^	5.6 ± 0.25^c^
0.25	81^a^	5.5 ± 0.21^a^	4.3 ± 0.18^d^
IBA			
0.025	57^d^	4.5 ± 0.14^b^	7.4 ± 0.31^b^
0.1	64^c,d^	5.0 ± 0.11^a^	9.5 ± 0.42^a^
0.25	69^c^	5.8 ± 0.23^a^	6.7 ± 0.28^b,c^

Values represent mean ± SE

Mean values followed by the same letters are not significantly different by the Duncan’s multiple range test at *p* = 0.05

After 4 weeks on multiple shoot induction medium, individual shoots were separated from each other and transferred to MS medium where they rooted within 2 weeks of culture (Fig. 2d). Over 100% of rooted plantlets survived when established in soil (Fig. 2e). The plants resumed growth in greenhouse reaching maturity and represented no phenotypic variation or sterility, irrespective of cultivars tested.

### Genotype influences on tissue culture response

It is known that the potential for multiple shoot induction and plant regeneration in rice depends on a number of factors of which genotype of the donor plant and interaction between genotype and shoot proliferation medium are most important. Therefore, in the present study eight *indica* rice cultivars were screened to evaluate the genotype influence on shoot proliferation and plant regeneration. The study showed the sensitivities of different *indica* rice cultivars to the optimal shoot proliferation medium. The shoot apices of cultivars IR-64, Mahasuri, and Nilagiri showed significantly greater shoot proliferation response than the other five cultivars on MS medium containing 4 mg/l TDZ (Table 3). However, no significant variation in regeneration frequency as well as mean shoot number was detected among the five cultivars, Ranjit, Vandana, Luit, Anjali, and Chandana (Table 3). Although, shoot apices *indica* rice cultivars IR-64, Mahasuri, and Nilagiri showed significantly greater shoot proliferation response, other five cultivars also responded to multiple shoot proliferation. Regeneration of different *indica* rice cultivars on the medium showed the technique is genotype-independent. Although a large number of protocols are available for embryogenic calli-mediated plant regeneration in *indica* rice, they are mostly genotype-dependent and furthermore, no universal medium adaptable to a number of *indica* rice genotypes has been developed. Furthermore, multiple shoots developed directly from the meristem without an intervening callus stage in the present protocol is expected to maintain genotype fidelity that could be lost with shoots arising from callus.

**Table 3:** Shoot multiplication and plant regeneration from shoot apices of eight cultivars of *Oryza sativa* spp. *indica* after 4 weeks of culture on MS medium containing 4 mg/l TDZ

Cultivar	Regeneration (%)	Mean shoot number	Average shoot length (cm)
IR-64	90^a^	9.3 ± 0.59^a^	7.1 ± 0.43^b^
Anjali	64^c^	7.9 ± 0.36^b^	6.3 ± 0.43^b^
Vandana	73^b,c^	2.1 ± 0.25^e^	4.2 ± 0.30^c^
Chandan	65^c^	4.3 ± 0.20^c,d^	6.4 ± 0.29^b^
Mahasuri	81^b^	4.0 ± 0.44^d^	9.1 ± 0.27^a^
Nilagiri	86^a^	5.5 ± 0.28^c^	9.6 ± 0.34^a^
Ranjit	76^b^	4.5 ± 0.26^c^	8.5 ± 0.54^a^
Luit	87^a^	5.0 ± 0.14^c^	4.3 ± 0.44^c^

Values represent mean ± SE

Mean values followed by the same letters are not significantly different by the Duncan’s multiple range test at *p* = 0.05

### Shoot apex transformation and *gus* expression

**Fig. 3:**
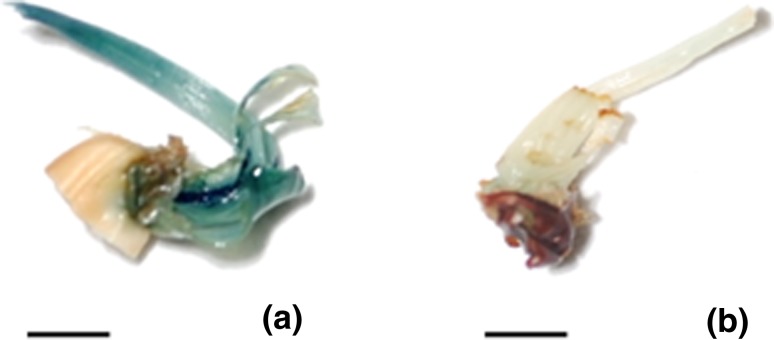
Transient GUS expression at the regenerating sites of shoot apex explants of *Oryza sativa* spp. *indica* cv. IR-64 after 3 days of co-cultivation. **a***Agrobacterium*-cocultivated shoot apex explants (transformed) after 3 days of co-cultivation **b** Control (untransformed). *Bar* represents 1 mm

**Fig. 4:**
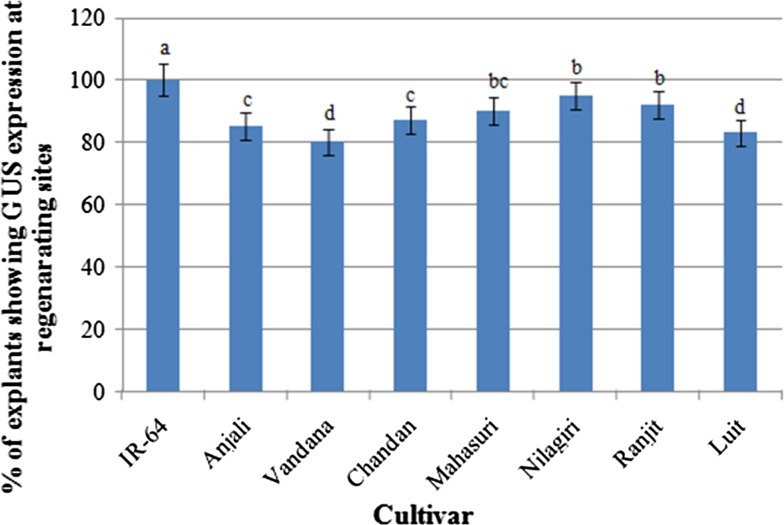
Transient GUS expression in shoot apex explants of different cultivars of *Oryza sativa* spp. *indica* after 3 days of co-cultivation. *X* axis showing rice cultivar use in transformation and *Y* axis showing % of *gus* expression at regenerating sites

It is generally known that genotype remains the major limiting factor restricting successful transformation in *indica* rice (Ge et al. 2006). The shoot meristem based plant regeneration system is mostly genotype-independent and provides a potential target for T-DNA delivery by *Agrobacterium* and direct gene transfer method (Sticklen and Oraby 2005). Another major advantage using the shoot apex explants is that rapid regeneration of shoots can be achieved from transformed shoot apices unlike shoot regeneration from transformed calli and protoplasts which involve several rounds of subculture involving risk of generating mutations (Arockiasamy and Ignacimuthu 2007). To determine the competency of the shoot apex explants of eight *indica* rice cultivars to *Agrobacterium*-mediated genetic transformation, experiments were carried out to inoculate explants with *A. tumefaciens*. 3 days after coculture, explants were incubated with substrate for β-glucuronidase enzyme and assayed for *gus* expression. Strong transient *gus* expression was detected in the region of the shoot apices from where the shoots developed, i.e., apex region (Fig. 3a). The endogenous GUS activity (color) was not detected in non-transformed (control) explants (Fig. 3b). GUS activity at the regenerating sites indicated the amenability of explants to *Agrobacterium*-mediated transformation. Although, shoot apices of all the *indica* rice cultivars showed strong *gus* expression at the regenerating site, however, transient *gus* expression efficiency differed from cultivar to cultivar (Fig. 4). Highest transient *gus* expression efficiency was recorded in cultivar IR-64 in which 100% of shoot apex explants showed *gus* expression (Fig. 4). Our results demonstrated that shoot apex explants of eight *indica* rice cultivars are amenable to *Agrobacterium*-mediated transformation and combined with their shoot proliferation ability could lead to regeneration of stable transgenic plants in a genotype-independent fashion. Transformation of the shoot apex is advantageous as a region of the shoot apex differentiates into the germline, which gives rise to the seed enabling transfer of the trait to the progeny. Shoot apices in conjunction with *Agrobacterium*-mediated transformation have been used to develop transgenic plants in two *indica* rice cultivars of Indian origin, WP and PB1 (Arockiasamy and Ignacimuthu 2007). Shoot apex-based regeneration systems have also been employed successfully to recover stably transformed maize, wheat, oat, barley, sorghum, and millet (Sticklen and Oraby 2005) and finger millet (Antony Ceasar and Ignacimuthu 2011).

### Molecular analysis of transgenic plants

**Fig. 5:**
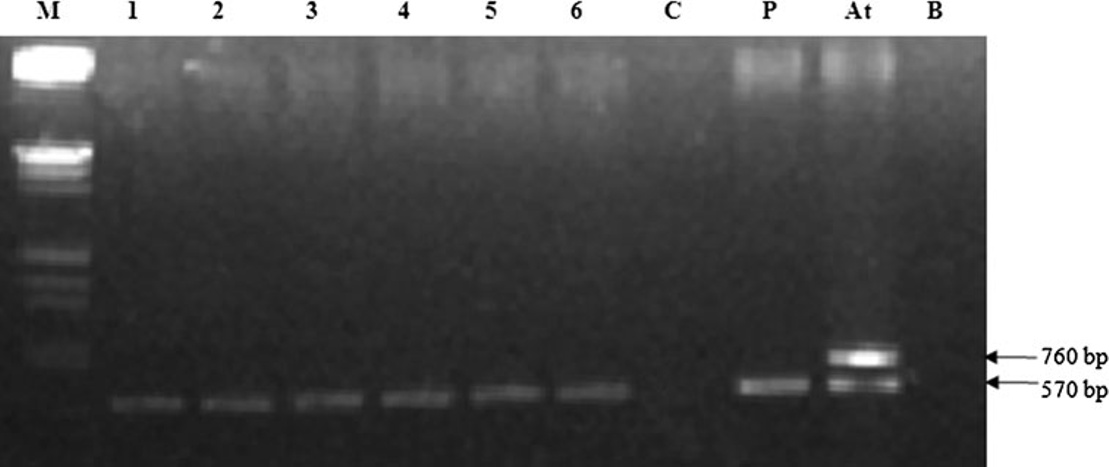
PCR screening for *virG* and *gus* genes on transformed plants developed from shoot apex of *Oryza sativa* spp. *indica* cv. IR-64. *Lane M* λDNA/*Eco*RI + *Hin*dIII marker, *lane P* pCAMBIA2301*Atpyl13* (positive control), *lane C* DNA from untransformed plant (negative control), *lane At* EHA105pCAMBIA2301*Atpyl13* (positive control for *virG* gene)*, lanes 1–6* DNA from independently transformed plants

The PCR analysis detected the presence of the expected 570 bp amplified product corresponding to *gus* (Fig. 5) in transformed shoots. No amplification was detected in the control untransformed shoots. Furthermore, the absence of signals in PCR by using *virG* primer in transformed shoots ruled out artefacts caused by *A. tumefaciens* contamination (Fig. 5).

## Conclusion

In conclusion, we have established rapid multiple shoot induction and efficient plant regeneration method from shoot apices of eight *indica* rice cultivars in a genotype-independent manner. The system was found amenable to *Agrobacterium*-mediated transformation in all the eight *indica* rice cultivars included in the study as evident from *gus* expression in transformed shoot apices and presence of *gus* gene in transformed plants by PCR analysis. This simple, rapid, and efficient plant regeneration system amenable to *Agrobacterium*-mediated transformation of eight *indica* rice cultivars may accelerate varietal development program through transgenic approach by incorporation of key candidate genes and for study of gene function.
